# Shyness and School Engagement in Chinese Suburban Preschoolers: A Moderated Mediation Model of Teacher–Child Closeness and Child Gender

**DOI:** 10.3390/ijerph19074270

**Published:** 2022-04-02

**Authors:** Yunpeng Wu, Min Fang, Jianfen Wu, Yingmin Chen, Hui Li

**Affiliations:** 1 School of Teacher Education, Dezhou University, Dezhou 253023, China; fangmin633@163.com; 2 Jing Hengyi School of Education, Hangzhou Normal University, Hangzhou 311121, China; 3 School of Psychology, Shandong Normal University, Jinan 250358, China; cc8030306@163.com; 4 School of Education, Faculty of Arts, Macquarie University, Sydney, NSW 2109, Australia; philip.li@mq.edu.au; 5 Shanghai Institute of Early Childhood Education, Shanghai Normal University, Shanghai 200234, China

**Keywords:** shyness, teacher–child relationship, school engagement, child gender, moderated mediation model, preschoolers

## Abstract

Shyness is associated with poorer preschool engagement, but few studies have evaluated the underlying mechanisms in Chinese preschoolers. This study explored the mediating role of teacher–child closeness and the moderating role of child gender in the association between shyness and school engagement to fill this gap. With the cluster sampling method, a total of 532 young children (240 girls; M_age_ = 4.29 years, SD = 0.65 years) were recruited from 15 suburban kindergartens in East China. Mothers rated children’s shyness, and teachers evaluated children’s school engagement and teacher–child closeness five months later. The results reveal the following: (1) Shyness was related to higher cooperative participation and lower school avoidance; (2) Teacher–child closeness mediated the relationships between shyness and school engagement. Specifically, shyness negatively predicted teacher–child closeness, and teacher–child closeness positively predicted cooperative and independent participations and school liking, and negatively predicted school avoidance; (3) Child gender moderated the relationship between shyness and school engagement, and specifically, for boys but not girls, shyness was significantly linked with lower school avoidance; for girls but not boys, shyness was significantly related to higher cooperative participation and lower independent participation. These findings have implications for the school engagement of preschoolers.

## 1. Introduction

Children’s positive emotional and behavioral engagement is beneficial for their school adjustment and their later learning and academic progress [1,2]. Investigating the influential factors of preschool engagement would help us to understand how to better prepare young children to transition to preschool, a novel and challenging group learning environment. In particular, shyness is a relevant personal characteristic that might influence preschoolers’ socialization in such a niche.

Shyness refers to the wariness in the face of social novelty or self-conscious behavior in perceived social evaluation situations [3]. The social novelty nature of the preschool classroom and potential social evaluation cues may hinder preschoolers’ emotional and behavioral engagement in kindergarten. Therefore, it is critical to understand the impact of shyness on preschool engagement and the underlying mechanism. In addition, teacher–child closeness, which reflects the warmth and open communication between children and their teacher, is an important interpersonal factor that may influence children’s preschool engagement.

In regard to the effect of the teacher–child relationship on the shyness–school engagement relationship, a Chinese study examined the moderating effects of gender and teacher–child relationships on the association between shyness and school adjustment [4]. However, the teacher–child relationship might also play a mediating role in the association between shyness and preschool engagement. This mediation effect needs to be examined empirically with Chinese preschoolers. This study aims to fill this gap by surveying the parents and teachers of a large sample of preschoolers in suburban areas of China.

### 1.1. Indicators of (Pre-)School Engagement

School engagement is receiving increasing research attention as educators contemplate solutions to school-related problems, and behavioral, emotional, and cognitive engagement have been identified as three important forms of school engagement [1]. Cognitive engagement generally refers to the level of processing or intellectual effort students devote to mastering learning tasks. As learning is not a preschoolers’ primary activity, cognitive engagement was not examined in the current study, and we focused on emotional and behavioral engagement.

The concept of emotional engagement refers to a student’s feelings about school and has been operationalized as children’s feelings about their peers, teachers, and schoolwork, or their affective reactions to the classroom or the larger school context [5,6]. As our focus in the current study, school liking and avoidance can be conceptualized as the degree to which children exhibit a receptiveness toward school. Specifically, school liking refers to a mindset that favors school and a desire to approach a school. In contrast, school avoidance refers to the intention to reject school and the desire to avoid school [5].

Behavioral engagement refers to participation in the learning environment and has often been operationalized in terms of how constructively or cooperatively children engage in classroom tasks and activities [7,8]. In this regard, we focused on two typical types of participation. Cooperative participation refers to children’s propensity to accept the teacher’s authority and comply with classroom rules and responsibilities. In contrast, independent participation refers to the extent to which children take the initiative or seek out and display self-directed behavior in the classroom [5]. Extant knowledge is also limited in that investigators tend to study single rather than multiple forms of engagement; thus, we investigated these four indicators separately in the current study.

### 1.2. Shyness and (Pre-)School Engagement

Shyness refers to wariness and anxiety in the face of social novelty and perceived social evaluation, despite a desire to interact socially [9]. Several similar concepts should be noted to deepen our understanding of shyness. First, social disinterest or unsociability, which are also social withdrawal phenomena, are driven by a low approach motivation [10,11], while shyness is characterized by the conflict between high approach and high avoidance motivation. In other words, a shy person intends to participate in social interactions, yet their anxiety and hesitation restrain them from doing so [9]. Second, the concept of behavioral inhibition emphasizes biologically based wariness in a novel context, whereas shyness has been defined as social wariness [3]. Third, two culture-specific concepts, shyness–sensitivity and regulated shyness, have been investigated in the Chinese culture. Shyness–sensitivity was defined as wariness and anxious social reactivity [12]. Regulated shyness, which refers to an acquiescent, nonassertive, and unassuming disposition during peer interaction, has been related to positive outcomes among Chinese children [13,14].

In the current study, shyness refers to the conflicted shyness defined by Coplan and colleagues, emphasizing feelings of internalized conflict (i.e., social fear and anxiety despite a desire to interact socially) [9]. A culturally equivalent construct allows for more valid cross-cultural comparisons. Meanwhile, focusing on the concepts theoretically and empirically related to negative social adjustment would benefit interventions for the potential negative consequences of shyness.

With regard to emotional engagement, the transition from home to the school environment and functioning in the classroom might be more difficult for shy children than non-shy children, and this might affect their affective connectedness to school [15,16]. This transition brings new peers and teachers, along with increased social and academic expectations. As shy children are reactive to novelty and experience excessive self-consciousness, such circumstances are stressful and might cause pressure to them. However, empirical support for relationships between shyness and school liking is limited. Shyness has been negatively related to the broad measure of school adjustment (a composite which included school liking) among preschoolers [17]. In addition, the teacher-rated shyness of children has been negatively related to concurrent child-reported school liking [18]. However, the direct relationship between shyness in kindergarten and school liking in second grade was not found, yet an indirect negative effect of shyness on school liking through popularity was found [19]. Discomfort in the classroom is expected to result in shy children showing low school liking.

The relationship between shyness and school avoidance has seldomly been investigated. However, shyness is not necessarily positively related to school avoidance. Possible negative attention/evaluation from parents and teachers, which may cause school avoidance intention or behavior, may drive shy children to reduce their school avoidance behaviors.

Concerning the relationship between shyness and behavioral engagement, the findings are mixed, and participation was generally examined as a composite index. For example, kindergartners’ shyness was found to be negatively related to their classroom participation, a composite index that included both cooperative and independent participation behaviors [18]. In addition, a cross-sectional study with a sample of 9- to 13-year-old children indicated that shyness was negatively related to academic engagement [16]. However, a three-year longitudinal study, which investigated cooperative participation, indicated that kindergarten shyness positively predicted second-grade cooperative participation [19]. The inconsistency of previous research indicates that aspects of shy children’s classroom engagement should be examined separately. Investigating these two forms of classroom participation as separate variables, rather than a composite one, would provide a clearer understanding of how shyness relates to classroom participation.

With regard to conceptual differences between cooperative and independent participation, their relationships with shyness are theoretically varied. Cooperative participation emphasizes compliance and cooperation. Prior research with Canadian elementary teachers indicated that shy children were perceived as non-disruptive, attentive listeners. However, they lacked confidence, facing difficulty in gaining peer acceptance, and intending to conform to classroom routines [20]. In addition, their social anxiety, which is likely to be triggered when interacting with authority figures (e.g., teachers), might also enhance their compliance [21].

As initiative and self-directed behavior are key features of independent participation, shy children’s lack of self-confidence and behavioral inhibition may hinder their independent participation in the classroom. In addition, experiencing anxiety may lead shy children to inhibit their speech and behavior in the classroom environment [15,22].

### 1.3. The Mediating Role of Teacher–Child Closeness

Despite evidence that shyness is generally related to poor school engagement, the mechanisms underlying this relationship have seldomly been investigated. To our knowledge, only one longitudinal study supported the mediating role of popularity with peers in the relationship between shyness and school, as well as the relationship between shyness and cooperative participation [19]. The teacher–child relationship, as another important aspect of children’s interpersonal relationships, needs to be investigated.

The quality of children’s relationships with teachers may influence their school engagement. Closeness, conflict, and dependency are three aspects of the teacher–child relationships, among which closeness is an important dimension. The warmth and open teacher–child communication embodied in the close teacher–child relationship is concurrently and predictively related to children’s positive school outcomes [23,24,25]. For the relationships between teacher–child closeness and emotional engagements, the teacher’s close relationship offers warmth and emotional connection. As for behavioral engagement, teachers can serve as a secure base from which children can explore the classroom and interact with their peers [26].

Besides the relationship between teacher–child closeness and school engagement, it was also found that shyness and teacher–child closeness are related. Teachers are more likely to report a less close relationship with shy children [27], and shyness was associated with less student-reported closeness in both Chinese and Dutch students [28]. We sought to extend these findings to evaluate a mediation model wherein we hypothesized that teacher reports of teacher–child closeness would mediate the relationship between shyness and school engagement.

### 1.4. The Moderating Role of Gender

Given that shy children in China may experience elevated adjustment difficulties, it is important to identify underlying factors that may interact with shyness, as they may also help in the design of prevention and intervention programs for shy children. Of particular interest for the current study is the role of child gender.

The gender differences in the developmental outcomes of shyness have been supported by studies carried out in Western and Chinese contexts. From the perspective of gender role stereotype theory, which casts males as traditionally more dominant/assertive and females as more passive/submissive [29], being shy is supposed to be more strongly associated with maladjustment among boys than girls, as shy behaviors may be perceived as violating male gender norms. A recent review of gender differences in childhood shyness, which was based on studies in Western cultural settings, suggested that shy behavior may put boys at increased risk for maladjustment as compared with girls, yet this assertion remains tentative in nature [30]. For example, a study on U.S. infants showed that fearful low-active (features of shyness) boys in the first year of life tended to show an increase in depressive symptoms, whereas fearful low-active girls showed a decline in depressive symptoms over time [31].

The findings pertaining to the effects of gender in the shyness literature among Chinese children are somewhat inconsistent. Shyness has been found to be more strongly associated with socio-emotional maladjustment among boys than girls in older Chinese children [32,33]. However, these findings have been somewhat mixed [4,34,35]. For example, in a study on the relationship between shyness and school adjustment with a Chinese urban preschooler sample, girls’ shyness, but not boys’, was related to higher cooperative participation [4]. Given the mixed data regarding the effect of child gender, we intend to examine the moderating role of child gender on the relationship between shyness and school engagement.

### 1.5. The Role of Social–Cultural Context

The social–cultural context also influences the relationships between shyness and adjustment. In China, unlike in Western culture, shyness has often been associated with positive adjustments, such as school competence and academic achievement, in third- to fifth-grade students in rural schools [34] and rural-to-urban migrant children [36]. In urban children, shyness was associated with positive social and school adjustment in the early 1990s, but has been associated with poor adjustment (peer rejection, school problems, and depression) since 2000 [12]. A study with Chinese urban and rural adolescents indicated that shy adolescents perceived more peer exclusion in the urban context [35].

The differences in sociocultural values have been proposed to explain the inconsistency in findings between China and Western countries and between urban and rural areas of China [37]. Independence, assertiveness, and autonomy are highly valued in individualistic cultures, thus shy behaviors in such cultural contexts are likely to be viewed as immature and weak. However, in collectivistic cultures, which value interdependence and group harmony, shy children are less likely to be viewed negatively, because they exhibit the intention to interact with others (though they show deficits in such interactions).

China has been characterized as more collectivistic and less individualistic than Western countries [38]. However, in different geographic areas of China (i.e., urban vs. suburban vs. rural), the sociocultural contexts vary due to varying effects of Western and economic influence. Contemporary urban samples in China were more individualistic compared with rural samples due to the dramatic social changes during globalization [39,40]. Compared with rural areas, where more traditional Chinese values have been retained, the urban areas that have experienced rapid economic growth have embraced more individualistic ideologies (e.g., independence, assertiveness) [12]. The suburban areas of China possess a unique context, mixing traditional and modern lifestyles and values [41].

Few studies have investigated how suburban children might be affected by shyness. Previous findings in other contexts may not apply to individuals in suburban areas. To our knowledge, only one study has investigated the links between shyness and adjustment in the suburban Chinese context, recruiting middle childhood and early adolescence participants; this study indicated that shyness tended to be related to social and psychological problems, more evidently in adolescence than in childhood [42]. This indicated that the implications of shyness may vary across individuals’ developmental periods. As for young children in suburban areas, a recent study with a preschooler sample indicated that unsociability, which is also a social withdrawal phenomenon, but different from shyness in its underlying drive, was positively associated with peer exclusion, asocial behavior, and anxious–fearful behavior [43], which indicated the maladjustment of unsociability in a suburban preschooler sample. However, as unsociability is different from shyness in nature, whether these findings applied to shyness should be examined. Thus, it is essential to investigate the implication of shyness in the preschooler population in the suburban context.

### 1.6. The Current Study

Although previous studies explored the correlations between shyness and school engagement, few examined the underlying mediating and moderating mechanisms among Chinese young children, especially those in suburban areas. The primary goal of the current study was to examine the potential mediating role of teacher–child closeness and the moderating role of child gender on the relationship between shyness and school engagement among Chinese suburban preschoolers. Overall, shyness was expected to be negatively related to school liking, school avoidance, and independent participation, and positively related to cooperative participation.

To examine the transactional processes underlying shyness and school engagement, based on the literature review, we hypothesized that the relationship between shyness and school engagement would be mediated by teacher–child closeness. That is, shyness was expected to be negatively related to teacher–child closeness, which in turn was expected to be related to higher school liking, higher cooperative participation, higher independent participation, and lower school avoidance.

In addition, we speculated that child gender moderates the relationship between shyness and school engagement. To be specific, we speculated that the effect of shyness on school liking, cooperative participation and independent participation for girls is stronger than that for boys, while the effect of shyness on school avoidance for girls is weaker than that for boys.

## 2. Methods

### 2.1. Sampling

To increase the external validity of the study in a time- and cost-efficient way, we applied the multistage cluster-sampling method.

First, we selected three third-tier cities (Jining, Heze, and Dezhou) in Shandong province of China. In China, with regard to indicators such as the size of urban built-up areas, the size of the urban population, the level of economic development and total GDP, cities are unofficially divided into three or more tiers. The first-tier cities are super-large cities such as Beijing, Shanghai, Shenzhen and Guangzhou. The second-tier cities are generally the capital or well-developed cities in each province. The third-tier cities are major cities in each province.

Second, we randomly selected 5 suburban kindergartens in each city. The suburban area is a certain area outside the built-up area of the city, and is influenced by the economic radiation, social and ideological penetration and urban ecological effects of the urban area. Despite its relatively poor economic development, it is closely linked to the economic development, lifestyle and ecosystem of the urban area.

Third, in each kindergarten, we randomly selected one class in each grade, resulting in three different grade classes in each kindergarten. There are normally three grades in a Chinese kindergarten: (1) Lower Kindergarten Class for children aged 3–4; (2) Middle Kindergarten Class for children aged 4–5; and (3) Upper Kindergarten Class for children aged 5–6.

A total of 45 classes of 15 suburban kindergartens participated in the current study.

### 2.2. Measures

#### 2.2.1. Shyness

Children’s shyness was assessed with the shyness subscale of the Chinese version of the Child Social Preference Scale (CSPS), which displayed satisfactory reliability and validity in the Chinese preschooler population [44]. Mothers rated seven items (e.g., “My child seems to want to play with others, but is sometimes nervous”) on a 5-point scale from 1 = definitely does not apply to 5 = definitely applies. Scale score was computed as a sum of all items; therefore, higher scores indicate higher shyness.

#### 2.2.2. Teacher–Child Closeness

We applied the Chinese version of the Student–Teacher Relationship Scale (STRS) [45] in the current study. This measure is of adequate reliability and validity in China [46]. We used the closeness subscale in the current study. Teacher rated 11 items (e.g., “I share an affectionate, warm relationship with this child”) assessing teacher–child closeness on a 5-point scale (from 1 = definitely does not apply to 5 = definitely applies). Scores were the sums of all items.

#### 2.2.3. School Engagement

School liking and avoidance were measured by the School Liking and Avoidance Questionnaire (SLAQ) [47]. Teachers reported the child’s school liking (5 items, e.g., “Enjoys school activities or events”) and school avoidance (4 items, same response scale, e.g., “Makes up reasons to stay home from school”) with a 5-point scale ranging from 1 = almost never applies to 5 = almost always applies.

To measure cooperative and independent participation, the cooperative participation subscale (7 items, e.g., “accept responsibility for a given task”) and the independent participation subscale (4 items; e.g., “works independently”) of the Teacher Rating Scale of School Adjustment (TRSSA) were applied. Teachers rated items on a 5-point scale ranging from 1 = definitely does not apply to 5 = definitely applies [48].

Scale scores represent the sums of item responses within each subscale, with a higher score indicating higher levels.

To confirm the validation of the measures, the Cronbach’s alphas and the indicators of convergent validity with confirmatory factor analysis were used (Table 1). The results indicate that all Cronbach’s alphas met the acceptable standard of more than 0.7, indicating the measurements had good reliability. For the validity of measurement, Fornell and Larcker’s standards suggest that composite reliability should exceed 0.06, and average variance extracted (AVE) should exceed 0.5 under ideal conditions, but 0.36~0.5 are acceptable [49]. Thus, the convergent validity was acceptable.

### 2.3. Procedure

This study was approved by the local ethics committee. The inclusion criteria (children aged 3–6 years; children in regular education classrooms) and exclusion criteria (children participating in any intervention study; children who are not living with parents) were set before the sampling. The mothers and homeroom teachers of those 45 sampled classes were enrolled and provided written consent. In Chinese kindergartens, each class typically has one or two homeroom teachers, responsible for handling children’s daily routines, keeping them physically safe, and teaching. They are also the head instructors of their classes, who take care of the same group of children during the kindergarten period. All participants were informed that their participation would be voluntary and they were free to withdraw their participation from this study at any time. Meanwhile, the anonymous nature of the study was emphasized before data collection.

The data were collected at two time points over one fall semester (T1: September 2020, the start of the semester; T2: January 2021, the end of the semester; five months interval).

- At T1, we collected the demographic information of children and their parents. Mothers rated their children’s shyness with CSPS. A total of 540 preschoolers from 45 classes of 15 suburban kindergartens participated in T1.
- At T2, the 45 homeroom teachers (age range: 19–44 years) of those 45 participating classes in T1 rated four indicators of school engagement for each participating child in their classes with TRSSA and SLAQ, then evaluated their closeness with each child with STRS. Their work experience in kindergarten ranged from 1.5 to 14 years. Eight preschoolers of T1 were lost due to a change in school or family residence, resulting in a final sample of 532 preschoolers.

Demographic information of the final sample is listed in Table 2.

### 2.4. Statistical Analyses

Data analyses were performed using IBM SPSS for Windows (version 26) and the macro-program PROCESS 4.0 of Hayes [50]. Before the data analysis, data cleaning was performed and a mixed linear model was then used to examine the classroom intraclass correlations in order examine whether there was significant variance due to grouping.

First, descriptive statistics and correlation analysis were used to analyze the questionnaire scores. Next, the mediating effect of teacher–child closeness was tested using Hayes SPSS macro PROCESS (Model 4). Then, the moderating effect of child gender on the direct link between shyness and school engagement was tested with SPSS macro PROCESS (Model 5). Four school engagement indicators (i.e., independent participation, cooperative participation, school liking, and school avoidance) were examined separately, resulting in 4 regression models.

The bootstrapping method with robust standard errors was applied to test the significance of the effects [50]. The bootstrapping method produced 95% bias-corrected confidence intervals (CIs) of these effects from 5000 resamples of the data. If CIs did not include zero, the effects in Model 4 and Model 5 were significant at α = 0.05. When evaluating moderator effects, researchers suggested transformations to z scores for interpretive purposes [51]. Thus, all predictors were standardized prior to the computation of interaction terms to avoid statistical artifacts associated with ulticollinearity, and no issues with tolerance were encountered.

As child gender is a dichotomous variable, the simple slopes were plotted using its categorical level (boy, girl). As children’s competencies develop and they become more familiar with the kindergarten environment, their school engagement is supposed to improve. Thus, the grade was controlled in the analysis.

## 3. Results

### 3.1. Descriptive Statistics and Correlation Analysis

Classroom intraclass correlations were less than 0.04 and nonsignificant for all variables, indicating no cluster effects for the classrooms in the present study.

Descriptive statistics and inter-correlations among the main study variables are listed in Table 3. Child gender (boys = 0, girls = 1) was significantly and positively associated with shyness, school liking, and cooperative participation while being negatively associated with school avoidance, indicating that girls showed a higher level of shyness, school liking and cooperative participation, and a lower level of school avoidance. The results indicate that shyness was negatively associated with school avoidance, whereas its correlations with the other three school engagement variables were not significant. Shyness was negatively associated with teacher–child closeness. In addition, teacher–child closeness was positively associated with school liking, independent participation, and cooperative participation, while being negatively associated with school avoidance.

### 3.2. Testing for Direct Effects

The results (Table 4) show that the total effect of shyness on school avoidance was significant and negative (β = −0.120, *p* < 0.01). When adding the teacher–child closeness variable to the regressions, the direct effect of shyness on school avoidance remained significant and negative (β = −0.141, *p* < 0.01). In addition, the direct effect of shyness on cooperative participation was significant and positive (β = 0.117, *p* < 0.01).

### 3.3. Testing for Mediating Effects

**Figure 1:**
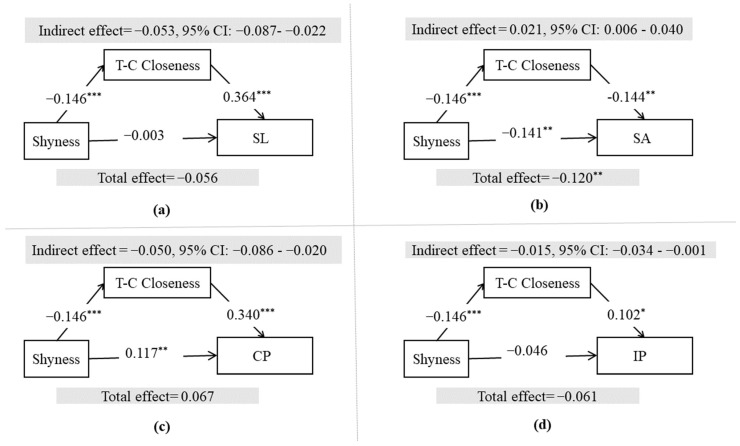
Mediation models with path coefficients. Note. The effects of grade was controlled. T-C = teacher–child, SL = school liking, SA = school avoidance, CP = cooperative participation, IP = independent participation. * *p* < 0.05; ** *p* < 0.01; *** *p* < 0.001. (**a**) Mediation model for school liking; (**b**) Mediation model for school avoidance; (**c**) Mediation Model for cooperative participation; (**d**) Mediation Model for independent participation.

We expected teacher–child closeness to mediate the relationships between shyness and school engagement. The results support the mediation hypothesis. The results indicate that shyness was negatively associated with teacher–child closeness (β = −0.146, *p* < 0.001). Meanwhile, teacher–child closeness was significantly and positively associated with school liking (β = 0.364, *p* < 0.001), cooperative participation (β = 0.340, *p* < 0.001), and independent participation (β = 0.102, *p* < 0.05), and significantly and negatively associated with school avoidance (β = −0.144, *p* < 0.01). The results of the bootstrap test (Figure 1) indicate that all 95% CIs did not include zero. According to Shrout and Bolger’s proposal, this indicated that the mediational effects were significant [52]. Thus, the mediating hypothesis was supported.

### 3.4. Testing for Moderating Effects

We expected that child gender would moderate the direct effect of shyness on school engagement. In the moderated mediation analysis (Table 5), the interaction between shyness and gender was significant and positive in terms of school avoidance (β = 0.239, *p* < 0.01) and cooperative participation (β = 0.205, *p* < 0.01); the interaction between shyness and gender was marginally significant and negative in terms of independent participation (β = −0.152, *p* < 0.1).

**Figure 2:**
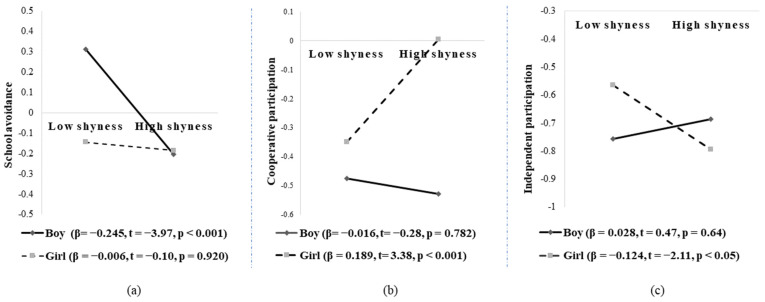
(**a**) Interaction between shyness and gender in predicting school avoidance; (**b**) interaction between shyness and gender in predicting cooperative participation; (**c**) interaction between shyness and gender in predicting independent participation.

In order to better comprehend the essence of the moderating effect of child gender, we plotted the simple slopes. As presented in Figure 2a, shyness was negatively and significantly linked with school avoidance for boys (β = −0.245, t = −3.97, *p* < 0.001), whereas the relationship was not significant for girls (β = −0.006, t = −0.10, *p* = 0.920). As presented in Figure 2b, shyness was positively and significantly linked with cooperative participation for girls (β = 0.189, t = 3.38, *p* < 0.001), whereas the relationship was not significant for boys (β = −0.016, t = −0.28, *p* = 0.782). As presented in Figure 2c, shyness was negatively and significantly linked with independent participation for girls (β = −0.124, t = −2.11, *p* < 0.05), whereas the relationship was not significant for boys (β = 0.028, t = 0.47, *p* = 0.64).

## 4. Discussion

This study proposed and examined a moderated mediation model as the first exploration of the mediating effect of teacher–child closeness as an explanatory mechanism underlining pathways from shyness to school engagement, and examined the moderating role of gender on the relationship between shyness and school engagement among a Chinese suburban preschooler sample. Most of our hypotheses were supported. The results indicate that (1) shyness was directly related to higher cooperative participation and lower school avoidance; (2) shyness was also indirectly linked to poor school engagement through its negative relationship with teacher–child closeness; specifically, shyness was negatively related to teacher–child closeness, whereas teacher–child closeness was positively related to cooperative participation, independent participation and school liking, and negatively related to school avoidance; and (3) the direct link between shyness and school engagement was moderated by child gender. This section will discuss these results, their implications, limitations, and future directions.

### 4.1. The Direct Effect of Shyness on School Engagement

Our first aim was to examine the direct effect of preschooler’s shyness on their school engagement among Chinese suburban preschoolers. The results indicate that shyness was related to higher cooperative participation and lower school avoidance. These novel results, which suggest the school-related benefits of shyness, refreshed our impression of shy children’s school engagement. The interpretation of these results should take the cultural context of the suburban area into consideration. Suburban areas, especially of the third-tier cities, though influenced by rapid economic development, still possess traditional collectivistic cultures which value interdependence and group harmony [41]. The compliance and nonassertive behaviors of shy children may account, at least partially, for their better school engagement.

The findings from the present study indicate that shyness does not necessarily lead to poor school-related outcomes. The longitudinal study also found that shyness in kindergarten predicted higher cooperative participation two years later [19]. Cooperative participation encompasses compliance, cooperation, and responsibility, whereas school avoidance contains a mindset that rejects school and behaviors related to avoiding school. Shy children’s desire to avoid negative attention/evaluation from teachers, parents, or peers may drive them to participate cooperatively in the classroom and reduce their school avoidance.

### 4.2. The Mediating Role of Teacher–Child Closeness

Our second aim was to examine the mediating role of teacher–child closeness. The findings of the current study indicate that shyness was indirectly linked to poor school engagement through its negative relationship with teacher–child closeness. For the first stage of the mediation process, shyness was negatively related to teacher–child closeness. A previous study indicated a possible reason for this relationship. A study of Italian preschoolers revealed that shyness predicted less social play, which in turn was related to less closeness with teachers [53]. For the second stage of the mediation process, teacher–child closeness was positively related to cooperative and independent participation and school liking, and negatively related to school avoidance

With regard to school avoidance and cooperative participation, two inconsistent mediation models were found. Inconsistent mediation models are models where at least one mediated effect has a different sign than the other mediated or direct effects in a model [54]. To be specific, the indirect effect of shyness through the mediating effect of teacher–child closeness was inconsistent with the direct effect of shyness.

These are interesting findings in that the indirect effect of shyness, via the mediation of teacher–child closeness, may suppress the direct relationship between shyness and cooperative participation, as well as the direct relationship between shyness and independent participation. First, shyness directly predicted higher cooperative participation. However, its indirect effect through teacher–child closeness on cooperative participation was negative, and the correlation between shyness and cooperative participation was not significant. Without taking teacher–child closeness as the mediating variable, the relationship between shyness and cooperative participation would be suppressed. Second, the direct effect of shyness on school avoidance was negative. However, the indirect effect of shyness was positive and significant. The coefficient between shyness and school avoidance was increased after taking teacher–child closeness into consideration. Shy children are typically viewed as compliant and well-behaved in the classroom [55]. However, their deficit in building a close relationship with their teachers may hinder their cooperative participation, and might drive them to avoid school.

With regard to school liking and independent participation, the mediating effects of teacher–child closeness were also found. Shyness was associated with low teacher–child closeness, which, in turn, was related to lower independent participation and school liking. A prior study indicated that shy children might have trouble with self-initiated behavioral involvement and show lower school liking [16,18]. The current study revealed a possible underlying mechanism. Teachers can serve as a secure base from which children can explore the classroom and interact with their peers [7,28]. A less close teacher–child relationship, as an indicator of shy preschoolers’ poor emotional connection with teachers, has a negative effect on their school liking and independent participation. With lower closeness with teachers, shy children may not feel secure enough to independently participate in the classroom, especially as children’s awareness of others’ evaluations and self-consciousness develop. In addition, as an indicator of their emotional connection with teachers, teacher–child closeness was negatively related to children’s school liking.

### 4.3. The Moderating Role of Child Gender

Our third aim was to examine the moderating role of child gender, and the results partially support our moderating hypothesis. The moderating role of child gender found in the present study further strengthened the evidence supporting the gender role stereotype theory.

Specifically speaking, for boys but not girls, shyness was significantly linked with lower school avoidance. The teacher-rated school avoidance in the current study, to a large degree, reflected children’s explicit intention to avoid school. The behaviors of avoiding or refusing school are not welcomed in China by teachers or parents. For children, exhibiting such behaviors requires assertiveness and boldness, which are endorsed by boys according to the perspective of gender role stereotype theory [29]. The negative correlation between gender and school avoidance, in the current study, indicated that boys exhibited a higher level of school avoidance than girls. This might account for our finding that the negative relationship between shyness and school avoidance emerged only among boys.

Our findings indicate that, for girls but not boys, shyness was significantly related to higher cooperative participation. This is consistent with a prior study [4]. In addition, our results also indicate that, for girls but not boys, shyness was significantly related to lower independent participation. The higher cooperative participation and lower independent participation of shy girls were also consistent with the perspective of gender role stereotype theory, which casts females as more passive and submissive [29]. Thus, this stereotype might strengthen the relationship between shyness and higher cooperative participation and lower independent participation among girls.

### 4.4. Theoretical Implications

This study has some theoretical implications. First, we found school-related benefits of shyness in the suburban context of China, which also corroborates the importance of considering the cultural context in research. Second, we identified teacher–child closeness as one mechanism by which shyness may engender negative school engagement. To our knowledge, this study is the first article investigating the mediating role of the teacher–child relationship on the relationship between shyness and school engagement. Third, the evidence supporting gender role stereotype theory was strengthened by the moderating role of child gender discovered in this study. These results may develop our knowledge of the shyness–school engagement relationship among Chinese suburban preschoolers and provide several implications (e.g., building a warm and close teacher–child relationship, being gender-specific) for interventions to address school engagement problems in shy preschoolers.

### 4.5. Practical Implications

The findings of the current study contribute some educational implications for early childhood education practice in China. First, two positive correlates of shyness (higher cooperative participation and lower school avoidance) were identified in the current study. Praising their performances in these aspects (e.g., higher obedience to classroom rules, lower absence from school) may reinforce shy children’s positive school engagement.

Second, identifying teacher–child closeness as one mechanism by which shyness may engender negative school engagement is important because it can inform efforts to promote school engagement by building a warm and close teacher–child relationship. Identifying the child characteristics or environmental factors that foster shy children’s close relationship with their teacher may prevent poor school engagement. For instance, at the characteristic level, interventions that teach shy children social skills may reduce shyness and improve socially competent behaviors [56]. On the environmental level, offering shy children more opportunities to interact with teachers might also improve their closeness with their teachers.

Third, an intervention program for school engagement based on the association between shyness and school engagement would benefit from being gender-specific.

### 4.6. Limitations and Future Directions

Despite the current study filling a number of gaps in the literature, the findings must be interpreted in light of a few limitations. First, this is a cross-sectional study; thus, the data might not establish a sound cause–effect relationship. For example, in terms of our interpretation of the mediation effect, it is also possible that preschoolers who have poor school engagement develop a less close relationship with their teachers, which in turn, increases their shyness. Longitudinal studies are needed in the future to gain better insight into the order of effects.

Second, although the use of different reporters was adopted in the current study, specifically, mothers rated children’s shyness, and teachers rated their relationship with children and children’s school engagement, thereby reducing (but not eliminating) inflated relationships due to common-method bias. Thus, future studies with multiple sources or informants (such as peers, teachers, or parental reports) for each variable are needed to verify the present findings.

Third, some of the effect sizes in this study were small or modest. As a result, we should take care not to overstate the significance of these effects. Furthermore, a small effect size may indicate that other important variables were not included in the study. The role of the teacher–child relationship in shyness and school engagement was examined in this study. Other social ties were not included (e.g., parent–child relationships or peer relationships). For example, peer relationships may be another mediator of this relationship. Prior studies indicate that peer acceptance mediates the relationship between shyness and school adjustment among third- to seventh-grade Chinese students [57], and friendship quality mediated the association between shyness and psychological outcomes in fourth- to eighth-grade urban children [58]. Future research should focus on these relationships in order to better understand shy preschoolers’ social networks and how these interpersonal relationships influence their school engagement, both independently and interactively.

Fourth, this study was conducted in suburban areas of three third-tier cities of East China; thus, the results could not be generalized to represent all Chinese suburban children. Compared with the suburban areas of first-tier cities such as Shanghai, the suburban areas of third-tier cities are supposedly less affected by the individualistic culture. In addition, the current study was conducted in Shandong province, which is the cradle of Confucianism. Shandong province is deeply influenced by traditional Confucian ideology, which promotes a collectivistic culture. To verify the findings of the current study, future studies would benefit from a larger sample from more suburban areas in China.

## 5. Conclusions

In summary, although further research is needed, the current study found that shyness can be beneficial for school engagement among suburban preschoolers in China. Furthermore, teacher–child closeness can be an explanatory factor for the association between shyness and school engagement, through which shyness is negatively related to school engagement. In addition, there are gender differences in the relationship between shyness and school engagement.

## Figures and Tables

**Table 1:** The factor loading range, Cronbach’s α, composite reliability, and average variance extracted of constructs.

Construct	Number of Indicators	Factor Loading Range	Cronbach’s α	Composite Reliability	Average Variance Extracted
Shyness	7	0.701–0.802	0.883	0.885	0.525
Teacher–Child Closeness	11	0.620–0.728	0.886	0.884	0.411
School Liking	5	0.721–0.882	0.874	0.873	0.581
School Avoidance	4	0.724–0.803	0.833	0.834	0.557
Cooperative Participation	7	0.708–0.814	0.891	0.890	0.537
Independent Participation	4	0.724–0.803	0.833	0.834	0.557

**Table 2:** Demographic statistics.

Variables	Female	Male	Total
Children			
Grade: *n* (%)			
Lower	74	86	160 (30%)
Middle	106	129	235 (44%)
Upper	60	77	137 (26%)
Total	240 (45%)	292 (55%)	532 (100%)
Age (year): M (SD)	4.32 (0.70)	4.27 (0.61)	4.29 (0.65)
Parents			
Education level: *n* (%)			
Junior high school and below	138 (26%)	106 (20%)	244 (23%)
Senior high school or vocational school	208 (39%)	192 (36%)	400 (38%)
Three-year college	96 (18%)	133 (25%)	229 (22%)
University/Bachelor’s	85 (16%)	90 (17%)	175 (16%)
Master’s and above	5 (1%)	11 (2%)	16 (1%)
Total	532 (100%)	532 (100%)	1064 (100%)
Age (year): M (SD)	30.25 (3.21)	31.32 (4.05)	30.79 (3.85)
Teachers			
Education level: *n* (%)			
Senior high school or vocational school	4	0	4 (9%)
University/Bachelor’s	37	2	39 (87%)
Master’s and above	2	0	2 (4%)
Total	43 (96%)	2 (4%)	45 (100%)
Age (year): M (SD)	-	-	28.26 (7.42)
Work experience (year): M (SD)	-	-	5.33 (5.62)

**Table 3:** Means, standard deviations and correlation coefficients for the study variables.

	1	2	3	4	5	6	7
1. Gender	-	0.154 ***	0.083 ^†^	0.145 ***	−0.138 **	0.215 ***	0.042
2. Shyness		-	−0.155***	−0.064	−0.119 **	0.061	−0.070
3. Closeness			-	0.417 ***	−0.120 **	0.359 ***	0.187 ***
4. School liking				-	−0.529 ***	0.469 ***	0.376 ***
5. School avoidance					-	−0.203 ***	−0.180 ***
6. CP						-	0.383 ***
7. IP							-
M	-	14.41	37.73	20.80	8.15	25.75	12.37
SD	-	5.38	5.75	3.26	3.12	5.32	3.08

Note. ^†^ *p* < 0.10, ** *p* < 0.01; *** *p* < 0.001. Gender (male = 0, female = 1). CP = cooperative participation, IP = independent participation.

**Table 4:** Mediation analysis.

	DVs	Predictors	*R* ^2^	*F*	β	*SE*	t
Step 1	SL	Grade	0.087	25.05 ***	0.287	0.182	6.91 ***
		Shyness			−0.056	0.025	−1.34
	SA	Grade	0.034	6.17 ***	−0.033	0.180	−0.76
		Shyness			−0.120	0.025	−2.79 **
	CP	Grade	0.055	15.43 ***	0.227	0.301	5.37 ***
		Shyness			0.067	0.042	1.59
	IP	Grade	0.096	28.06 ***	0.302	0.171	7.30 ***
		Shyness			−0.061	0.024	−1.48
Step 2	Closeness	Grade	0.102	30.13 ***	0.280	0.044	−3.55 ***
		Shyness			−0.146	0.317	6.80 ***
Step 3	SL	Grade	0.206	45.69 ***	0.185	0.177	4.58 ***
		Shyness			−0.003	0.024	−0.06
		Closeness			0.364	0.023	8.90 ***
	SA	Grade	0.034	6.17 ***	0.007	0.186	0.17
		Shyness			−0.141	0.025	−3.26 **
		Closeness			−0.144	0.025	−3.18 **
	CP	Grade	0.159	33.21 ***	0.132	0.297	3.17 **
		Shyness			0.117	0.040	2.90 **
		Closeness			0.340	0.040	8.06 ***
	IP	Grade	0.105	20.70 ***	0.273	0.177	6.37 ***
		Shyness			−0.046	0.024	−1.11
		Closeness			0.102	0.023	2.34 *

Notes: *n* = 532. DV = dependent variable, β = standardized coefficients; SL = school liking, SA = school avoidance, CP = cooperative participation, IP = independent participation. * *p* < 0.05; ** *p* < 0.01; *** *p* < 0.001.

**Table 5:** Main and moderating effects of shyness and gender on indices of school engagement.

Predictors	SL		SA		CP		IP	
	β	t	β	t	β	t	β	t
Shyness	0.016	0.28	−0.245	−3.97 ***	−0.016	−0.28	0.028	0.47
Closeness	0.357	8.71 ***	−0.142	−3.16 **	0.317	7.64 ***	0.105	2.41*
Gender	0.211	2.67 **	−0.221	−2.54 *	0.330	4.11 ***	0.042	0.49
Shyness × Gender	−0.070	−0.90	**0.239**	2.79 **	**0.205**	2.58 *	**−0.152**	**−1.82 ^†^**
Grade	0.240	4.45 ***	0.019	0.32	0.165	3.01 **	0.364	6.33 ***

Note. All continuous variables were standardized before entering the regressions. SL = school liking, SA = school avoidance, CP = cooperative participation, IP = independent participation. ^†^ *p* < 0.10; * *p* < 0.05; ** *p* < 0.01; *** *p* < 0.001. All significant interaction effects are presented in bold.

## Data Availability

The data presented in this study are available on request from the corresponding author.
